# Metabolomic and Transcriptomic Profiling Uncover the Underlying Mechanism of Color Differentiation in *Scutellaria baicalensis* Georgi. Flowers

**DOI:** 10.3389/fpls.2022.884957

**Published:** 2022-06-09

**Authors:** Defu Wang, Jiangran Wang, Yufen Wang, Dongzuo Yao, Yanbing Niu

**Affiliations:** College of Life Sciences, Shanxi Agricultural University, Jinzhong, China

**Keywords:** anthocyanin, flowers, *S*. *baicalensis*, transcriptome, metabolic profiling

## Abstract

*Scutellaria baicalensis* Georgi. (Chinese skullcap or Huang-qin) is an extremely crucial medicinal plant in the Labiate family, and the color of its flowers naturally appears purple. However, during the long-term cultivation of *S*. *baicalensis*, very few plants of *S*. *baicalensis* also present white and purple-red flower colors under the same ecological conditions. However, the complex metabolic and transcriptional networks underlying color formation in white, purple-red, and purple flowers of *S*. *baicalensis* remain largely unclarified. To gain an insight into this issue, we conducted transcriptome and metabolomic profiling to elucidate the anthocyanin synthesis metabolic pathway in the flowers of *S*. *baicalensis*, and to identify the differentially expressed candidate genes potentially involved in the biosynthesis of anthocyanins. The results showed that 15 anthocyanins were identified, among which cyanidin 3-rutinoside and delphin chloride were the primary anthocyanins, and accumulation was significantly related to the flower color changes of *S*. *baicalensis*. Furthermore, the down-regulation of *SbDFR* (*Sb02g31040*) reduced the anthocyanin levels in the flowers of *S*. *baicalensis*. The differential expression of the *Sb3GT* (*Sb07g04780* and *Sb01g72290*) gene in purple and purple-red flowers affected anthocyanin accumulation, suggesting that anthocyanin levels were closely associated with the expression of *SbDFR* and *Sb3GT*, which play important roles in regulating the anthocyanin biosynthesis process of *S*. *baicalensis* flowers. Transcriptomic analysis revealed that transcription factors WRKY, bHLH, and NAC were also highly correlated with anthocyanin accumulation, especially for NAC35, which positively regulated *SbDFR* (*Sb02g31040*) gene expression and modulated anthocyanin biosynthesis in flower color variation of *S*. *baicalensis*. Overall, this study presents the first experimental evidence for the metabolomic and transcriptomic profiles of *S*. *baicalensis* in response to flower coloration, which provides a foundation for dynamic metabolic engineering and plant breeding, and to understand floral evolution in *S*. *baicalensis* plants.

## Introduction

*Scutellaria baicalensis* Georgi. (Chinese skullcap or Huang-qin) is an erect, perennial herb belongs to the Labiate family, which has been cultivated for its therapeutic properties in China for 2000 years (Shang et al., 2010; Zhao et al., 2016a,b). The dried root of *S*. *baicalensis* is rich in flavonoids and used extensively as a traditional medicine for treating fever and lung and liver complaints (Li, 2012). Modern pharmacological research shows that the bioactivity of root flavonoids from *Scutellaria* also has antibacterial, antiviral, antioxidant, anticancer, hepatoprotective, and neuroprotective properties (Baumann et al., 2008; Gao et al., 2011; Yang et al., 2012; Nayak et al., 2014).

The flower color of most *S*. *baicalensis* usually appears purple in nature and in the field, while our research group found that some plants of *S*. *baicalensis* have white and purple-red flowers under the same ecological conditions. Flower color is affected by various external and internal factors, but anthocyanin type and content are some of the most significant factors that may influence flower color. Anthocyanins, which are natural colorants belonging to an important subgroup of flavonoids, cannot only make plant tissues and organs show different colors but also help plants resist biological and abiotic stress, including protecting plants from pathogenic bacteria infection, resisting ultraviolet radiation, and removing excess active oxygen (Chen et al., 2012; Wang et al., 2013). Thus far, about 635 anthocyanins have been identified (He and Giusti, 2010). Cyanidin, delphinidin, pelargonidin, peonidin, petunidin, and malvidin are six common anthocyanins in the plant kingdom (Jaakola, 2013). Among them, delphinidin, malvidin, and petunidin are important coloring substances in many blue-purple plant organs; peonidin and cyanidin are the main pigments in purple-red plant organs, while pelargonin appears in brick red plant organs. These compounds have been reported to change the color of flowers from pink to blue violet (Tanaka et al., 2009; Peng et al., 2021). Anthocyanin biosynthesis is primarily associated with the pathway of phenylalanine metabolism (Dooner et al., 1991; Silva et al., 2007). The key enzymes involved in the earlier stages of anthocyanin synthesis are chalcone isomerase (CHI), chalcone synthase (CHS), flavonoid 3′-hydroxylase and flavanone 3-hydroxylase (F3H); while those in the later stages are anthocyanidin 3-glycosyltransferase, anthocyanidin synthase (ANS) and dihydroflavonol 4-reductase (DFR). At present, the genes responsible for anthocyanin biosynthesis have been found in *Ipomoea batatas* (Wang et al., 2013), *Ginkgo biloba* (Cheng et al., 2013), mulberry (Li D. et al., 2020), alfalfa (Duan et al., 2020), and other plants. It has been reported that color changes may occur in fruit, leaves and flowers, and the overexpression of enzymes-encoding genes and single-gene mutations can affect anthocyanin accumulation and color variation (Han et al., 2012; Xie et al., 2012; Li et al., 2013).

Apart from the above-mentioned biosynthetic genes, many transcription factors (TFs) are involved in the modulation of anthocyanin synthesis pathway and color changes (Saito et al., 2013). The roles of helix-loop-helix (bHLH), R2R3-MYB and WD40 have been widely investigated in recent years, which can form a MYB-bHLH-WD40 (MBW) complex to directly regulate the expression of genes related to anthocyanin synthesis pathway, thus promoting the biosynthesis of anthocyanins (Gonzalez et al., 2008; Suzuki et al., 2016; Bai et al., 2019; Chen et al., 2019). In addition, the formation of anthocyanins also depends on acylation, glycosylation, hydroxylation and methoxylation to improve stability, and some TFs play a crucial role in the transport of anthocyanins in plants (Lou et al., 2014; Wu et al., 2016).

Recently, a combination of high-throughput approaches has been proposed to assess color changes. The integrated analysis of metabolomic and transcriptomic data have revealed the altered secondary metabolites and differentially expressed genes (DEGs) in flowers and fruit (Wang et al., 2017; Dong et al., 2019; Meng et al., 2019; Zhuang et al., 2019; Li H. et al., 2020; Zhang et al., 2020), thus providing a global view of plant color development. The present study aimed to explore the transcriptional and metabolic profiles of three different flower colors in *S*. *baicalensis*, and the DEGs that responded to anthocyanin biosynthesis, three cultivars of *S*. *baicalensis* with different flower colors (SbP, SbPR, and SbW) were employed. Transcriptional, metabolic, and integrative analyses were conducted on three *S*. *baicalensis* plants with different flower colors. The findings provide important insights into the underlying mechanism of anthocyanin accumulation and the anthocyanin metabolic pathway in *S*. *baicalensis* flowers, which lay a substantive foundation for future molecular and functional studies in the creation of new *S*. *baicalensis* germplasm with different petal colors to enrich agricultural landscape planning and design.

## Materials and Methods

### Plant Materials

Three kinds of *S*. *baicalensis* with different petal colors were cultivated at the *S*. *baicalensis* germplasm resource center at Shanxi Agricultural University, China, and were termed ‘purple petals’ (SbP), ‘purple-red petals’ (SbPR), and ‘white petals’ (SbW). Fresh purple, purple-red, and white petals were harvested from healthy *S*. baicalensis at the same development stage in July 2020. All materials were snap-frozen in liquid nitrogen and stored at –80°C for metabolite extraction and RNA sequencing. All experiments in this study were conducted using three replicates.

### Measurement of Relative Anthocyanin Content

After the plants of *Scutellaria* blossomed, 0.1 g of petal of the corresponding groups were collected, ground with 1 mL methanol (0.1% HCl), rinsed for two times, and placed in a 10-mL centrifuge tube. The samples were eluted with methanol (0.1% HCl) to a final volume of 5 mL. The tissue homogenate was oscillated for 30 s, followed by centrifugation (12,000 g, 10–15 min, 4°C). The supernatant extract was filtered with a 0.22-μm inlet filter, and the optical density of the supernatants was detected using an ultraviolet spectrophotometer at 530 nm. The relative levels of anthocyanins were calculated as follows: Q = V × A_530_/M (units/g FW), where V and M represent the volume of the solution and the weight of the samples. The concentration of anthocyanin at OD_530_ = 0.1 was regarded as 1 unit to calculate the relative content of anthocyanin in the samples. Methanol (0.1% HCl) was employed as a blank control (Jiang et al., 2020).

### Sample Preparation for Metabolomic Analysis

The vacuum freeze-dried *S*. *baicalensis* specimens were ground to powder using a mixer mill (MM 400, Verder Retsch, Shanghai, China) at 30 Hz for 90 s. During extraction, 100 mg of powder was weighed and dissolved in 1 mL aqueous methanol (70%). Subsequently, the mixture was incubated overnight at 4°C in a refrigerator. During incubation, the samples were swirled three times at 10-min intervals (10 s, 40 Hz) to improve the extraction rate. After centrifugation (10,000 g, 10 min), the extract was absorbed using a CNWBOND Carbon-GCB SPE Cartridge (250 mg, 3 mL; ANPEL, Shanghai, China)^1^ and filtrated (SCAA-104, 0.22-μm pore size; ANPEL, Shanghai, China)(see text footnote 1) prior to ultra-performance liquid chromatography-tandem mass spectrometry (UPLC-MS/MS) analysis.

### Ultra-Performance Liquid Chromatography Conditions and ESI-Q TRAP-MS/MS

The extracted samples were subjected to LC-ESI-MS/MS analysis (UPLC, Shim-pack UFLC SHIMADZU CBM30A^2^, MS/MS, Applied Biosystems 4500 Q TRAP^3^) (Chen et al., 2013). The following conditions were used: ACQUITY UPLC HSS T3 (C18) columns (Waters; 2.1 mm × 100 mm, 1.8 μm). The mobile phase was composed of solvent A (ultra-pure water with 0.1% formic acid) and solvent B (acetonitrile). The following gradient program was applied: 0 min V(A)/V(B) (100:0), 11.0 min V(A)/V(B) (5:95), 12.0 min V(A)/V(B) (5:95), 12.1 min V(A)/V(B) (95:5), and 15.0 min V(A)/V(B) (95:5). The injection volume, column oven temperature and flow rate were 5 μL, 40°C, and 0.4 mL/min, respectively. Mass spectrometric detection was performed in electrospray ionization (ESI) positive mode. The effluents were connected alternatively to an ESI-QTRAP-MS/MS. The standards were detected with a gas temperature of 550°C, capillary voltage of 5500 V, and nebulizer pressure of 25 psi.

### Identification and Quantification of Metabolites

The metabolites were identified and quantified by the Wuhan MetWare Biotechnology Co., Ltd. (Wuhan, China). The scheduled multiple reaction monitoring (MRM) method previously described by Fraga et al. (2010) was used. Metabolites were identified using the MetWare MWDB database and public database of MassBank^4^, KNAPSAcK^5^, HMDB^6^, MoTo DB^7^, and METLIN^8^ (Wishart et al., 2013; Zhu et al., 2013). The filtering conditions for the differentially accumulated metabolites (DAMs) were as follows: absolute log2 (fold-change) ≥ 1, variable importance in projection (VIP) ≥ 1 and *p*-value < 0.05. To determine the specific DAMs, R software^9^ was employed to conduct principal component analysis (PCA) and orthogonal partial least squares-discriminant analysis (OPLS-DA). Pathway enrichment analysis on differential metabolites was performed using the Kyoto Encyclopedia of Genes and Genomes (KEGG) (Zhuang et al., 2019).

### RNA Extraction, Illumina Sequencing, and Annotation

TRIzol reagent (Invitrogen, Carlsbad, CA, United States) was applied to extract total RNA from different flowers by following the kit’s protocol. The RNA concentration and purity of each sample were determined by NanoDrop ND-1000 (NanoDrop Technologies, Wilmington, DE, United States). Meanwhile, the RNA integrity was assessed by the Agilent Bioanalyzer 2100 system (Agilent Technologies, Santa Clara, CA, United States). mRNA was enriched from the total RNA using magnetic beads with oligo (dT) primers, and cDNA was synthesized by SuperScript™ II Reverse Transcriptase (Invitrogen, cat. 1896649, Carlsbad, CA, United States) and linking the sequencing adapter to both ends. The libraries were then sequenced on an Illumina Novaseq™ 6000 platform (LC-Bio Technology Co., Ltd., Hangzhou, China). Clean reads were extracted with base-pair qualities of Q ≥ 20 via custom Perl scripts, followed by mapping on the *S*. *baicalensis* genome using the HisAT2 software with default settings.

### Screening of Differentially Expressed Genes

The differentially expressed genes (DEGs) were selected with the thresholds of log2 (fold-change) ≥ 1 and false discovery rate (FDR) < 0.05 using the edge package in R (Robinson et al., 2010). Gene ontology (GO) enrichment analysis was implemented using the topGO package in R (corrected *p*-value < 0.05). The KEGG database^10^ was used to conduct pathway analysis (Kanehisa et al., 2008).

### Integrative Analysis of Transcriptomic and Metabolomic Profiles

The DEGs from anthocyanins and TFs responsible for the biosynthesis of anthocyanins were correlated with 15 DAMs of anthocyanins. Integrative analysis was conducted by calculating the FPKM values of genes and metabolites, and the screening criterion for DEGs was |r| > 0.8, and |r| > 0.9 and *p*-value < 0.05 for metabolites. Using an online tool (Lianchuan Cloud Biological Platform)^11^ to reveal the interactive networks among DEGs and DAMs of anthocyanins. A heatmap was drawn based on DEGs and 15 anthocyanins using R software.

### Quantitative Real-Time Polymerase Chain Reaction Assays

For use in gene expression validation of RNAseq, total RNA was isolated from *Scutellaria* flowers and analyzed by quantitative real-time polymerase chain reaction (qRT-PCR) protocols. Twelve genes associated with anthocyanin biosynthesis were randomly selected from RNAseq for qRT-PCR analysis, and the constitutively expressed *actin* was employed as a housekeeping gene. All specific primer pairs are listed in Supplementary Table 1. The 25-μL reaction mixture consisted of 1 μL first-strand cDNA, 20 μL 1 × SYBR Premix ExTaq (Takara), 1 μL forward primer (10 μM), 1 μL reverse primer (10 μM), and 2 μL highly pure water. The reaction was performed in an ABI 7500 fast (ABI, Fort Lauderdale, FL, United States). This experiment conducted in triplicate. The expression level of each target gene was determined by the 2^–△^^△^ *^Ct^* method using the *actin* gene as an internal standard (Schmittgen and Livak, 2008).

### Sequence Alignment, Phylogenetic Analysis, and Three-Dimensional Structure Prediction of the Candidate gene

Multiple amino acid sequence alignment was conducted with DNAMAN software (Lynnon Corp., Canada). Phylogenetic analysis was performed using MEGA 7.1 by the maximum likelihood method with 1,000 bootstrap replicates. The 3D structures of the candidate gene were predicted using the ITASSER V3.0 and V5.0 server.^12^ UCSF Chimera V1.9 program^13^ was used to visualize the protein data bank files retrieved from this server.

### Statistical Analyses

Statistical tests were conducted with Excel 2010 software (Microsoft Office, Redmond, WA, United States). All values are presented as mean ± standard deviation (SD). Least significant difference test was employed to compare the means between groups (*p* < 0.05).

## Results

### Total Anthocyanin Content in Three Different Flowers of *Scutellaria baicalensis*

**FIGURE 1:**
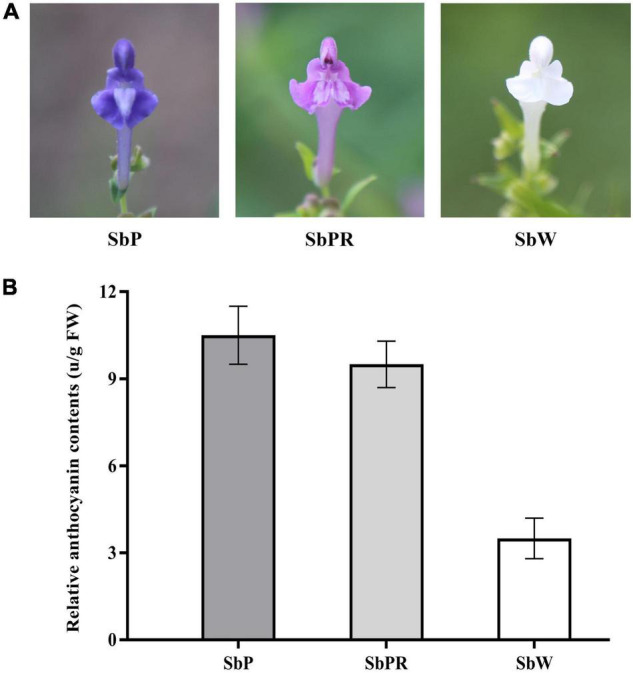
*Scutellaria baicalensis* Georgi. flowers used in this study. **(A)** Phenotypes of *S*. *baicalensis* flowers. **(B)** Measurement of relative anthocyanin contents in flowers of SbW, SbPR, and SbP. SbW, white flower at the blooming stage; SbPR, purple-red flower at the blooming stage; SbP, purple flower at the blooming stage.

Although *S*. *baicalensis* was grown in the same ecological environment, the petal colors in different plants were obviously different, with SbW possessing white petals, SbPR possessing purple-red petals, and SbP possessing purple petals (Figure 1A). Anthocyanins are the major pigments in plants. In this work, the anthocyanin content in three different *S*. *baicalensis* flowers was measured. The relative anthocyanin levels of SbP and SbPR were 10.5 and 9.5 units/g of fresh weight, respectively, which were remarkably higher compared with SbW (3.5 units/g of fresh weight, Figure 1B).

### Metabolite Analysis on Three Different Flowers of *Scutellaria baicalensis*

**FIGURE 2:**
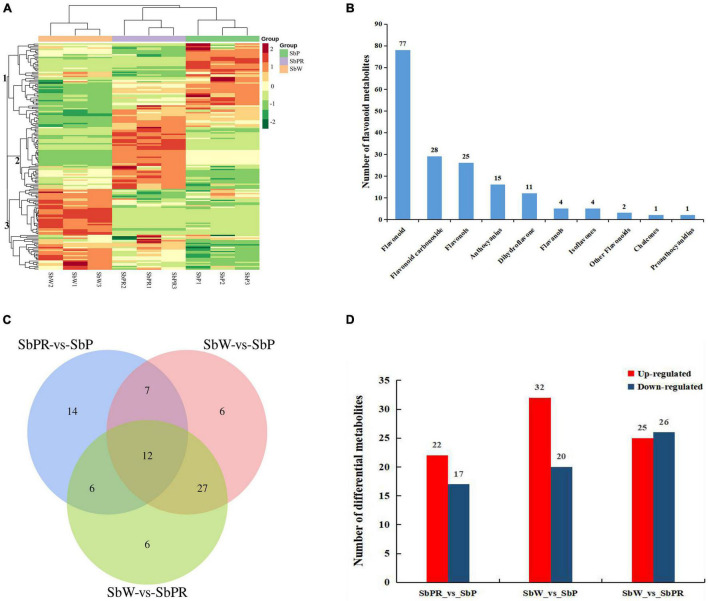
Flavonoid metabolome profiles of the petals of white, purple-red, and purple flowers in *Scutellaria baicalensis*. **(A)** Heatmap clustering showing the correlation among white, purple-red, and purple flower samples based on 168 metabolite profiles. The color scale from red to green indicates the normalized metabolite levels using the row Z-score. **(B)** Classification of the 168 identified metabolites into 10 groups. **(C)** Venn diagram indicating the shared and specific number of the differential metabolites identified by each package. **(D)** Numbers of differential metabolites. SbW: white flower at the blooming stage; SbPR, purple-red flower at the blooming stage; SbP, purple flower at the blooming stage.

Based on LC-MS widely targeted metabolome technology, qualitative and quantitative analyses of metabolites were conducted by MRM of triple quadrupole mass spectrometry. R software was used to construct a heatmap for metabolites. According to the differences in the accumulation of metabolites among different samples, hierarchical cluster analysis revealed that significant differences were found in metabolites among three different flowers of *S*. *baicalensis*, and three main clusters were obtained according to the relative differences of flavonoid accumulation patterns (Figure 2A). The flavonoids in clusters 1 accumulated at the highest levels in SbP followed by SbPR, and at the lowest levels in SbW. The flavonoids in cluster 2 were at the highest levels in SbPR followed by SbP, and were at the lowest levels in SbW. The flavonoids in cluster 3 were at the highest levels in SbW followed by SbPR, and were at the lowest levels in SbP. In total, 168 different flavonoid metabolites, such as 25 flavonols, 15 anthocyanins, 28 flavonoid carbonosides, 11 dihydroflavones, 4 flavanols, 4 isoflavones, 77 flavonoids, 1 chalcones, 1 proanthocyanidins, and 2 other flavonoids, were identified in the flowers of *S*. *baicalensis*. The details of all identified metabolites are summarized in Figure 2B and Supplementary Table 2.

To analyze the metabolic differences between SbW, SbPR, and SbP, screening of differential metabolites was conducted on all metabolites based on the following criteria: fold-change ≥ 1, *p*-value < 0.05 and VIP ≥ 1, which were graphed by Venn diagrams (Figure 2C). A total of 78 differential metabolites were identified among all samples and the relative contents of these differential metabolites were illustrated in a heatmap (Supplementary Figure 1). Among these differential metabolites, 12 differential anthocyanins were included, and their relative contents were described (Supplementary Figure 2). In addition, the overlapping metabolites were further identified. The results showed that 12 differential metabolites were shared among SbPR vs. SbP, SbW vs. SbP, and SbW vs. SbPR. These 12 differential metabolites included six flavonoids, two flavonols, one dihydroflavonols, and three anthocyanins. The three anthocyanins are classified as Cyanin chloride, Cyanidin 3-rutinoside and Delphin chloride. As demonstrated in Figure 2D and Supplementary Table 3, 39 differential metabolites (17 down-regulated and 22 up-regulated) were observed between SbPR and SbP, 52 differential metabolites (20 down-regulated and 32 up-regulated) between SbW and SbP, and 51 differential metabolites (26 down-regulated and 25 up-regulated) between SbW and SbPR. Subsequently, the differential anthocyanins among these samples were obtained by analyzing the relative anthocyanin quantification results (Supplementary Figure 3). The results indicated that there were 5 (3 down-regulated and 2 up-regulated), 11 (0 down-regulated and 11 up-regulated), and 10 (1 down-regulated and 9 up-regulated) types of differential anthocyanins in SbPR vs. SbP, SbW vs. SbP, and SbW vs. SbPR, respectively, and these might be the key metabolites that influencing petal coloration in *S*. *baicalensis* (Supplementary Figure 3).

A total of 15 anthocyanins, such as cyanidin, delphinidin, pelargonidin, peonidin and malvidin, were identified in *S*. *baicalensis* flowers. Among them, the content of cyanidin 3-rutinoside in SbPR and SbP was significantly higher than that in SbW (105947- and 6828-fold, respectively), which suggested that it was an important color substance in *S*. *baicalensis*. The cyanidin 3-rutinoside and delphin chloride contents in SbPR were 645-fold higher and 53.8-fold lower, respectively, than those in SbP (Table 1). The significant difference in cyanidin 3-rutinoside and delphin chloride contents may be involved in the purple and purple-red flower colors of *S*. *baicalensis*.

**TABLE 1:** Differential accumulation of anthocyanins in the flowers of *Scutellaria baicalensis* ‘white petals’ (SbW), ‘purple-red petals’ (SbPR), and ‘purple petals’ (SbP).

Component name		Content		Fold change	Variable importance in projection (VIP)
SbW_vs_SbP	Metabolite name	SbW	SbP	SbP/SbW	
	Cyanidin 3-rutinoside (Keracyanin chloride)	9.00E+00	6.15E+04	6.83E+03	3.01E+00
	Cyanin chloride	8.35E+05	1.99E+07	2.38E+01	1.80E+00
	Cyanidin 3-*O*-galactoside	1.48E+06	3.23E+07	2.19E+01	1.78E+00
	Delphin chloride	4.59E+06	5.41E+07	1.18E+01	1.58E+00
	Peonidin 3-*O*-glucoside chloride	8.73E+04	4.44E+05	5.09E+00	1.29E+00
	Cyanidin 3-*O*-malonylhexoside	8.49E+05	2.60E+07	3.06E+01	1.87E+00
	Cyanidin 3-*O*-glucoside(Kuromanin)	1.78E+06	3.98E+07	2.23E+01	1.79E+00
	Pelargonidin 3-*O*-malonylhexoside	2.84E+03	2.31E+04	8.12E+00	1.46E+00
	Cyanidin *O*-syringic acid	2.14E+05	2.45E+06	1.15E+01	1.58E+00
	Cyanidin *O*-acetylhexoside	1.11E+05	3.83E+06	3.46E+01	1.91E+00
	Centaureidin	9.54E+05	2.99E+06	3.14E+00	1.07E+00

**SbW_vs_SbPR**	**SbW**	**SbPR**		**SbPR/SbW**	

	Cyanidin 3-rutinoside (Keracyanin chloride)	9.00E+00	9.54E+05	1.06E+05	3.00E+00
	Cyanin chloride	8.35E+05	1.56E+08	1.87E+02	2.02E+00
	Cyanidin 3-*O*-galactoside	1.48E+06	4.55E+07	3.08E+01	1.64E+00
	Delphin chloride	4.59E+06	1.01E+06	2.19E-01	1.09E+00
	Peonidin 3-*O*-glucoside chloride	8.73E+04	6.32E+05	7.24E+00	1.24E+00
	Cyanidin 3-*O*-malonylhexoside	8.49E+05	6.39E+07	7.53E+01	1.84E+00
	Cyanidin 3-*O*-glucoside(Kuromanin)	1.78E+06	5.55E+07	3.12E+01	1.64E+00
	Pelargonidin 3-*O*-malonylhexoside	2.84E+03	5.22E+04	1.84E+01	1.51E+00
	Cyanidin *O*-syringic acid	2.14E+05	1.79E+06	8.36E+00	1.29E+00
	Cyanidin *O*-acetylhexoside	1.11E+05	9.36E+06	8.45E+01	1.86E+00

**SbPR_vs_SbP**	**SbPR**	**SbP**		**SbP/SbPR**	

	Cyanidin 3-rutinoside (Keracyanin chloride)	9.54E+05	6.15E+04	6.45E-02	1.73E+00
	Cyanin chloride	1.56E+08	1.99E+07	1.27E-01	1.50E+00
	Pelargonin chloride	1.78E+04	3.75E+03	2.10E-01	1.30E+00
	Delphin chloride	1.01E+06	5.41E+07	5.38E+01	2.08E+00
	Centaureidin	3.23E+05	2.99E+06	9.25E+00	1.56E+00

### Transcriptome Analysis of Three Different Flowers of *Scutellaria baicalensis*

**FIGURE 3:**
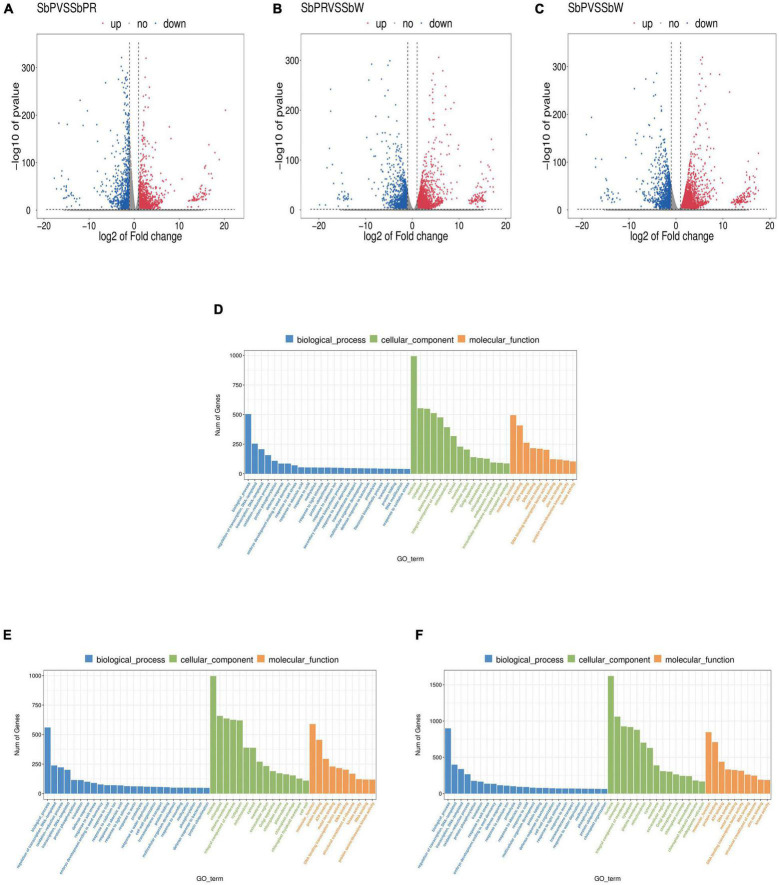
Transcriptome analysis of genes in SbW, SbPR, and SbP. **(A–C)** The volcano plot indicates the differential gene expression levels among SbW, SbPR, and SbP. Blue dots represent down-regulated DEGs; red spots represent up-regulated DEGs; and gray represents non-DEGs. **(D–F)** GO enrichment of the differential genes in SbW, SbPR, and SbP. SbW: white flower at the blooming stage; SbPR, purple-red flower at the blooming stage; SbP, purple flower at the blooming stage.

To clarify the molecular mechanism of anthocyanin synthesis in three different flowers of *S*. *baicalensis*, transcriptomic analysis was conducted to identify DEGs in three different flowers. RNA-seq produced 40,138,441, 44,209,405, and 41,126,941 clean reads from SbP, SbPR, and SbW libraries, respectively. Using the filter criteria |Log_2_FC| ≥ 1 and *P* < 0.05, there were 4,418, 4,898, and 7,378 DEGs in the three comparison groups: SbP vs. SbPR, SbPR vs. SbW, and SbP vs. SbW, respectively. Comparing the three different flowers of *S*. *baicalensis*, 3,165, 3,457, and 6,026 genes were up-regulated, while 1,253, 1,441, and 1,352 genes were down-regulated in SbP vs. SbPR, SbPR vs. SbW, and SbP vs. SbW, respectively (Figures 3A–C and Supplementary Table 4). Using GO enrichment analysis, DEGs were enriched in three GO groups: molecular functions, cellular components and biological processes (Figures 3D–F). The enrichment analysis of KEGG metabolic pathways of DEGs showed that they were associated with enrichment in metabolic processes, such as the flavonoid biosynthesis pathway and phenylalanine synthesis pathway (Table 2 and Supplementary Table 5).

**TABLE 2:** Significant enrichment in KEGG pathways among SbW, SbPR, and SbP.

No.	Pathway	DEGs with pathway annotation	All genes with pathway annotation	*P*-value	Pathway ID
**SbW_vs_SbP**					
1	Anthocyanin biosynthesis	3	23	0.99	ko00942
2	Flavonoid biosynthesis	38	112	0.26	ko00941
3	Phenylpropanoid biosynthesis	73	264	1.78E + 00	ko00940
4	Plant hormone signal transduction	132	572	1.00	ko04075
5	Starch and sucrose metabolism	118	360	0.21	ko00500
**SbW_vs_SbPR**					
1	Flavonoid biosynthesis	21	112	0.71	ko00941
2	Phenylalanine metabolism	18	92	0.63	ko00360
3	Protein processing in endoplasmic reticulum	59	363	0.98	ko04141
4	Anthocyanin biosynthesis	4	23	0.72	ko00942
**SbPR_vs_SbP**					
1	Starch and sucrose metabolism	59	360	0.69	ko00500
2	Plant hormone signal transduction	75	572	1.00	ko04075
3	Phenylpropanoid biosynthesis	59	264	0.02	ko00940
4	Flavonoid biosynthesis	29	112	0.01	ko00941
5	Anthocyanin biosynthesis	6	23	0.19	ko00942

### Integrated Transcriptomic and Metabolomic Analyses to Reveal Anthocyanin Synthesis in Three Different Flowers of *Scutellaria baicalensis*

**FIGURE 4:**
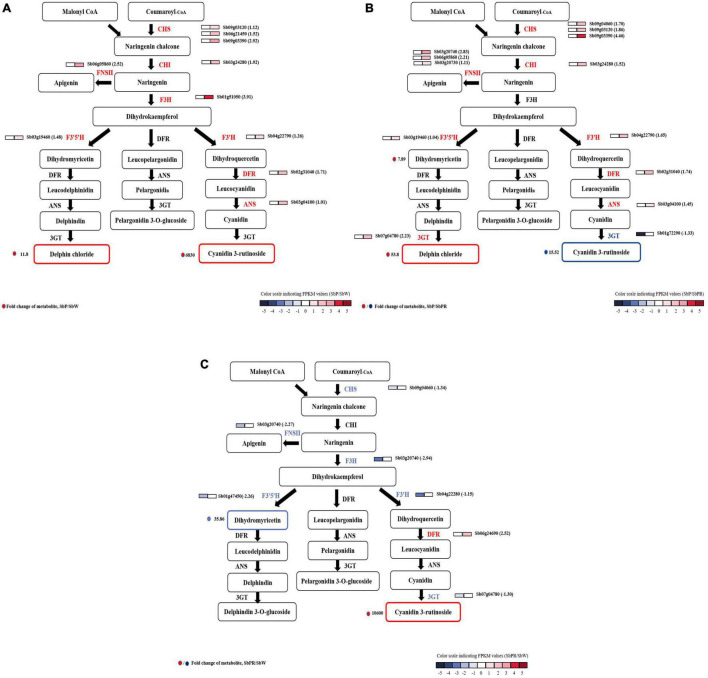
Transcript profiling of genes related to the flavonoid synthesis pathway with different flower colors (SbP, SbPR, and SbW). The core schematic illustration of flavonoid synthesis in *S*. *baicalensis* flowers was constructed according to KEGG pathway analysis and published literature [**(A)** SbP vs. SbW; **(B)** SbP vs. SbPR; **(C)** SbPR vs. SbW). The annotated flavonoid synthesis genes and the candidate genes for regulating anthocyanins synthesis in *S*. *baicalensis* flowers are shown in the figure with different colors. The grids with color-scale from dark to light indicate RPKM values; red and blue represent high and low abundances, respectively. Red or dark solid circles represent the log2 fold change in metabolites contents between three groups (SbP vs. SbPR, SbPR vs. SbW, or SbP vs. SbW). ANS, Anthocyanidin synthase; CHI, chalcone isomerise; CHS, chalcone synthase; DFR, dihydroflflavonol 4-reductase; F3′5′H, flavonoid-3′,5′-hydroxylase; F3H, flavanone 3-hydroxylase; FNS, flavone synthase; GT, UDP-glucosyltransferase.

By analyzing the single genes responsible for anthocyanin synthesis, the key enzyme genes of *S*. *baicalensis* with three different flowers were discovered. Through detailed comparative analysis, it was found that most secondary metabolite pathways were enhanced by the up-regulation of gene expression. In SbW vs. SbP, the expression levels of upstream (CHS, CHI, etc.) and downstream genes (DFR, 3GT, etc.) in the anthocyanin biosynthesis pathway were higher in SbP than in SbW. These genes were increased by 1.01- to 3.9-fold (Figure 4A). In SbPR vs. SbP, structural genes CHS, CHI, F3′H, ANS, and DFR increased 1.04 to 4.46-fold, and the Sb3GT gene (an up-regulated and down-regulated DEG) was up-regulated by 2.25-fold and down-regulated by 4.60-fold, respectively. Sb3GT can catalyze the modification of unstable anthocyanins in *S*. *baicalensis* and transform them into stable anthocyanins (Kobayashi et al., 2001), and the change in its expression may affect the accumulation of anthocyanins. This also supported the increase of metabolite delphin chloride and the decrease of cyanidin 3-rutinoside in SbP (Figure 4B). In SbPR vs. SbW, it was found that except for *DFR*, the expression level of other genes in SbPR was lower than that in SbW, but the anthocyanin level was higher in SbPR than in SbW (Figure 4C), which suggested that the down-regulated expression of *DFR* affected the formation of anthocyanins. In summary, differentially expressed *DFR* and *Sb3GT* genes can regulate the anthocyanin synthesis pathway in *S*. *baicalensis*.

### Correlation Analysis of Differentially Expressed Genes and Anthocyanins

**FIGURE 5:**
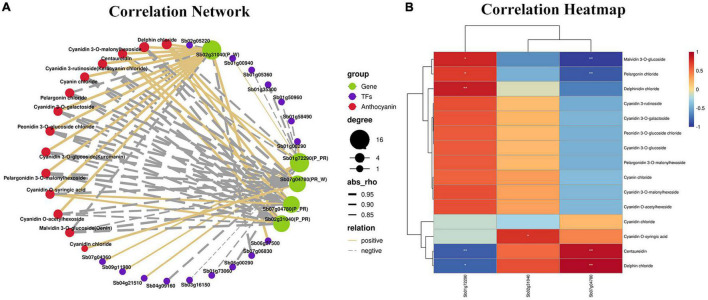
Connection network between metabolite and transcriptome profiles. **(A)** Connection networks among DEGs, TFs, and anthocyanin metabolites. The red, green, and blue colors represents anthocyanin metabolites, DEGs and TFs, respectively. The solid and dotted lines represent positive and negative correlations, respectively. Line width represents the degree of correlation. **(B)** Correlation heatmap of three DEGs in the anthocyanin synthesis pathway and 15 anthocyanins among the three different-colored flowers of *Scutellaria baicalensis*. The heatmap was constructed according to the FPKM value. The red and blue represent positive and negative correlations, respectively.

**FIGURE 6:**
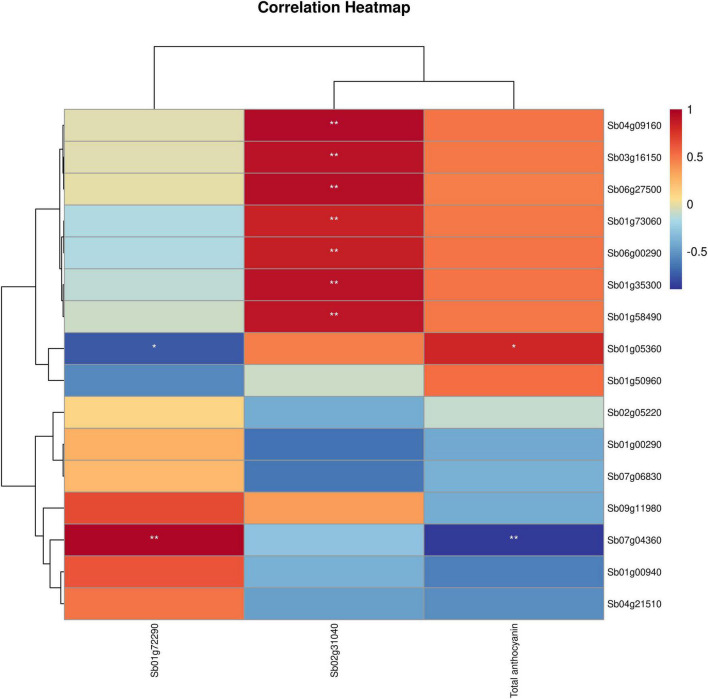
Correlation heatmap of TFs with total anthocyanins and key genes. The color scale on the right represents the degree of correlation among TFs, genes and anthocyanins. The red represents high correlation degree and blue represents low correlation degree. One asterisk represents correlation and two asterisks represent significant correlation.

To analyze the regulatory networks of anthocyanins and genes involved in different flower colors in *S*. *baicalensis*, three DEGs (*Sb02g31040*, *Sb07g04780*, and *Sb01g72290*) and 15 anthocyanins were selected as the source data for correlation network analysis and heatmap generation. As shown in Figure 5A, delphin chloride was highly correlated with *Sb02g31040*(*P_W*), *Sb01g72290*(*P_PR*), *Sb02g31040* (*P_PR*), *Sb07g04780*(*P_PR*), *and Sb07g04780*(*PR_W*), and they were all positively correlated except for *Sb01g72290* (*P_PR*). Cyanidin 3-rutinoside (Keracyanin chloride) was highly correlated with *Sb02g31040*(*P_PR*), *Sb07g04780*(*P_PR*), *Sb02g31040*(*P_W*), *and Sb07g04780*(*PR_W*), and they were all negatively correlated except for *Sb02g31040*(*P_W*) (Figure 5A). The transcription factors of DEGs were also identified in the correlation network, including WRKY, WD40, MYB, bHLH, and NAC. Among them, WRKY31, bHLH66, NAC100, and WRKY40 were positively correlated with *Sb01g72290* (*P_PR*), MYB, WD40, WRKY, NAC62, and WRKY53 were negatively correlated with *Sb01g72290* (*P_PR*), NAC79, and NAC2 were negatively correlated with *Sb02g31040* (*P_W*), NAC35 was positively correlated with *Sb02g31040* (*P_W*) (Figure 5A). Heatmap analysis showed that there were significant differences between selection DEGs and anthocyanins. Among them, the regulation extent of *Sb02g31040* and *Sb07g04780* to delphin chloride biosynthesis was significantly higher than that of *Sb01g72290*, which was in good agreement with the findings of the correlation network analysis (Figure 5B). Furthermore, by analyzing the correlation among anthocyanin content, transcription factors and key genes, we found an interesting phenomenon that TFs positively regulated *Sb01g72290* (*P_PR*) expression resulting in a decrease in anthocyanin accumulation, TFs negatively regulated *Sb01g72290* (*P_PR*) expression resulting in an increase in anthocyanin accumulation, and TFs positively regulated *Sb02g31040* (*P_W*) expression resulting in an increased anthocyanin accumulation (Figure 6 and Supplementary Table 6). In total, *Sb02g31040*, *Sb01g72290* and WRKY, bHLH, and NAC jointly participated in the regulation of the biosynthesis of delphin chloride and cyanidin 3-rutinoside among three different colored flowers of *S*. *baicalensis*.

### Key Genes Responsible for Anthocyanin Biosynthesis in Three Different *Scutellaria baicalensis* Flowers

**FIGURE 7:**
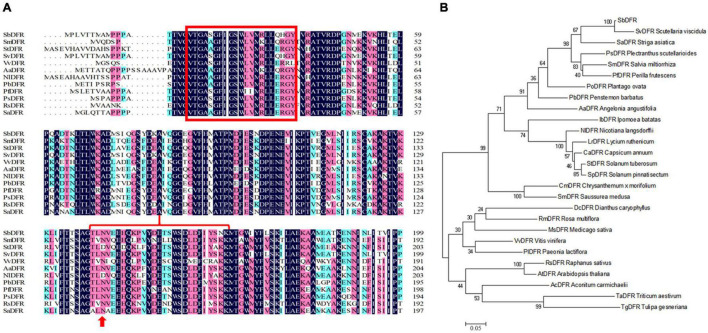
Phylogenetic and multiple alignment analyses of the deduced amino acid sequence of the SbDFR protein from *Scutellaria baicalensis* and other species. **(A)** Multiple alignment of the proteins from selected species. The NADPH binding domain and substrate specific binding domain are marked by the red box and red brace, respectively, and the site marked by the red arrow is the amino acid residue influencing enzyme substrate specificity. **(B)** Phylogenetic analysis of SbDFR proteins from *S*. *baicalensis* and other selected species.

**FIGURE 8:**
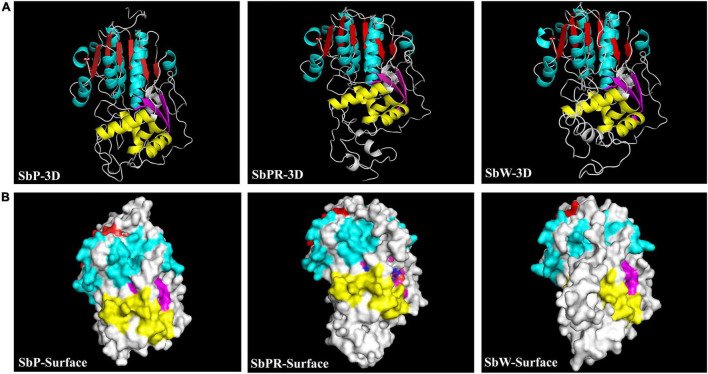
The predicted 3D protein structure of DFR in *Scutellaria baicalensis* flowers. **(A)** The 3D structural model of the full-length DFR in SbP, SbPR, and SbW. **(B)** Predicted surface localization corresponding to three DFR secondary structures were revealed by UCSF Chimera program. The same secondary structure in the same area is marked with the same color. The red and yellow marks represent the same secondary structure of α-helice in different functional areas, and the blue and purple marks represent the same secondary structure of β-turn in different functional areas.

Based on our transcriptome database analysis, *SbDFR* (Sb02g31040) and two differentially expressed *Sb3GT* genes (Sb07g04780 and Sb01g72290) were identified. BLAST analysis indicated that *SbDFR* has a high similarity (99.87%) with *SvDFR* (GenBank: FJ605512.1). The amino acid sequence analysis showed that the SbDFR protein had two conserved domains in the NADB-Rossmann superfamily, namely the NADPH binding domain and substrate-specific binding domain (Figure 7A). Phylogenetic analysis showed that it had the closest genetic relationship with *S*. *viscidula* (Figure 7B). To identify key domains for catalytic activity, I-TASSER program was applied to predict the 3D structure of the DFR protein. The results demonstrated that the DFR protein had two pairs of α-helices and six β-turns located at the N-terminal region (aa 1–260). The four predicted α-helices contained amino acids at 24–35, 98–125, 170–188, and 239–251, and six predicted β-turns located at 15–19, 38–45, 88–92, 130–140, 192–197, and 257–260 (Figure 8). The six β-corners and two pairs of α-helices form the Rossmann folding region, which is the binding site region of coenzyme NADP^+^ and DFR substrate specificity. In this study, at site 121, SbW had leucine, which is a hydrophobic amino acid, while SbPR and SbP had serine, which is a hydrophilic amino acid. At site 164, SbPR had lysine, and SbW and SbP had aspartic acid. These two different sites are located in the Rossmann folding structure, and their differences may lead to DFR selectively catalyzing substrates. Based on the 3D model analysis of its key domains, surface catalytically active protein regions are different, and the base mutation in this structural region may affect the change in catalytic substrate.

**FIGURE 9:**
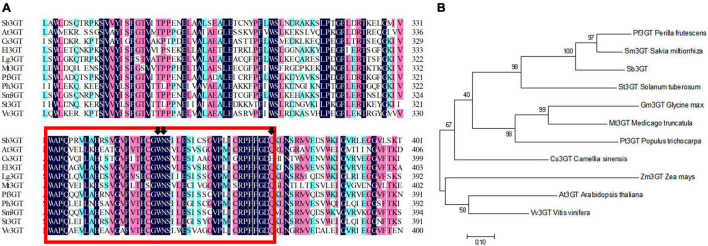
Phylogenetic and multiple alignment analyses of the deduced amino acid sequence of Sb3GT protein from *Scutellaria baicalensis* and other species. **(A)** Multiple alignment of the proteins from selected species. A N-terminal-conserved plant secondary product glycosyltransferases (PSPG) domain is framed in a red box. The black arrow indicates the significant amino acid sites of 22, 23, and 44 in the PSPG domain. **(B)** Phylogenetic analysis of the Sb3GT proteins from *S*. *baicalensis* and other selected species.

The Sb3GT belongs to the UDP-glucosyltransferase (UAGT) superfamily. The amino acid sequence analysis showed that Sb3GT (Sb07g04780) had a plant secondary product glycosyltransferase (PSPG) located at the N-terminal region (aa 333–376) (Figure 9A). Phylogenetic analysis showed that it had the closest genetic relationship with *S*. *miltiorrhiza* (Figure 9B). The sequence of *Sb3GT* (Sb01g72290) was not found in the NCBI databases. It is speculated that this gene is a novel *Sb3GT* gene in *S*. *baicalensis*.

The correlation between the expression level of these genes and total anthocyanin content was further calculated. The results showed that two DEGs positively regulated the anthocyanin synthesis, and one DEG negatively regulated anthocyanin synthesis. The expression levels of two DEGs, namely *SbDFR* (Sb02g31040) and *Sb3GT* (Sb07g04780), indicated a significant positive correlation with total anthocyanin content in the samples, suggesting that these two DEGs may have an essential role in anthocyanin accumulation (Supplementary Table 7).

### Transcription Factors Associated With the Accumulation of Anthocyanin

Transcription factors are involved in the biosynthesis of anthocyanins in plants by affecting target gene expression. By comparing SbP vs. SbW, SbP vs. SbPR, and SbPR vs. SbW, 127, 79, and 73 TFs-related DEGs, respectively, were identified and were mainly annotated as bHLH, MYB, WD40, WRKY, NAC, and MADS-box. These transcription factors may separate or form a complex to regulate the biosynthesis of anthocyanin metabolites in the three different flowers of *S*. *baicalensis* (Supplementary Table 8). Furthermore, by calculating the correlation between the expression levels of these TFs and total anthocyanins content, 81 TFs with remarkable correlation (|Cor| ≥ 0.5, *P* value < 0.05) were involved in the accumulation of anthocyanins were identified, including 13 negative regulator and 68 positive regulators. These negative regulators include 3 WRKY, 1 NAC, 5 MYB, and 4 MADS box, which likely act as a repressor in anthocyanin synthesis. The 68 positive regulators, including 3 WRKY (such as WRKY53), 11 WD40, 4 NAC (such as NAC35), 14 MYB, 5 MADS box, 1 bZIP, 11 bHLH, and 19 others, may act as promotors of anthocyanin accumulation (Supplementary Table 9).

### Validation of the Transcriptomic Data Through Quantitative Real-Time Polymerase Chain Reaction

**FIGURE 10:**
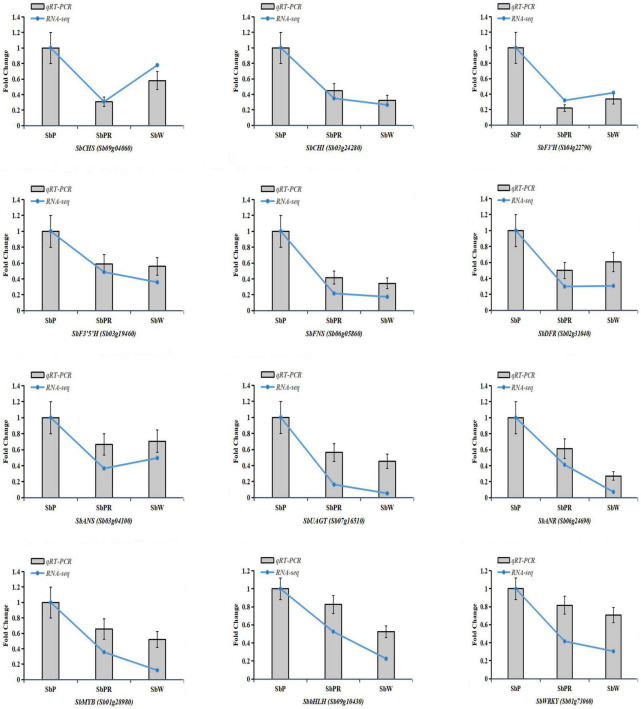
Verification of representative genes related to anthocyanin biosynthesis by qRT-PCR. Relative expression levels of candidate genes were calculated using the *actin* gene as a standard. Error bars represent the standard deviation of three replicates. Curve graphs represent gene expression from the RNAseq data based on FPKM. Bar charts represent the gene expression levels calculated from qRT-PCR data based on ΔCt method.

To evaluate the reliability of transcriptome information, 12 DEGs (3 transcription factor genes and nine flavonoid biosynthetic pathway genes) were chosen to validate the RNA-seq data. The qRT-PCR results indicated that the expression profiles of 12 selected DEGs were highly correlated with those obtained from the transcriptome data (Figure 10).

## Discussion

### Identification of Anthocyanin From the Flowers of *Scutellaria baicalensis*

Anthocyanins, as important secondary metabolites, widely exist in flowers, fruit, leaves, and seed coats and participate in many physiological and biochemical reactions of plants (Liu et al., 2001; The Angiosperm Phylogeny Group, 2009; Dong et al., 2011; Brunetti et al., 2013; Mierziak et al., 2014; Grimes et al., 2018). In the present study, to explore anthocyanin synthesis pathway in white, purple, and purple-red flowers of *S*. *baicalensis*, transcriptomic and metabolomic analyses were conducted. Based on UPLC-MS, 168 metabolites were identified, including 15 anthocyanins. The analysis of anthocyanin differential metabolites in SbP, SbPR, and SbW showed that the content of cyanidin 3-rutin (chlorokeratin), reported as the major anthocyanins in the peels of “Brown Turkey”, “Bursa”, and “Black Mission” figs (Solomon et al., 2006; Ercisli et al., 2012), was 105,947- and 6,828-fold higher in SbPR and SbP than in SbW, respectively (Table 1). The cyanidin 3-rutinoside (keracyanin chloride) content was 15.52-fold higher in SbPR vs. SbP, while the content of delphin chloride in SbPR was 53.78-fold lower than in SbP. This result suggests that cyanidin 3-rutinoside and delphin chloride are the key anthocyanins conferring pigment accumulation in flowers of *S*. *baicalensis*. Interestingly, this study found that there were six common anthocyanin pigments (cyanidin, delphinidin, pelargonidin, petunidin, peonidin, and malvidin) present in *S*. *baicalensis* flowers. The decisive factors of flower color may be related to the number of pigment molecules, metallic ions and/or various molecular conformations of anthocyanins. The pigment molecules accumulated to different degrees in *S*. *baicalensis* flowers, which induced the biosynthesis of anthocyanins with different colors in the metabolic pathway.

### Genes Involved in Anthocyanin Biosynthesis

Through transcriptome sequencing analysis and the identification of single genes involved in the biosynthesis of anthocyanins, CHI, CHS, FNSII, F3′H, F3H, F3′5′H, 3GT, and DFR were expressed to different degrees in three different flower colors of *S*. *baicalensis*. In SbPR vs. SbP, the structural gene *Sb3GT* in the anthocyanin synthesis pathway was differentially expressed, while in SbPR vs. SbW, other significant structural genes except the *SbDFR* gene were down-regulated. Studies have found that the expression of enzymes-encoding genes is involved in the regulation of anthocyanin synthesis (Saito et al., 2013). For example, high expression of *ANS*, *DFR* and anthocyanidin 3-*O*-glucosyltransferase (*UFGT*) can affect color changes in fruit (Han et al., 2012). The down-regulated expression levels of *DFR* and *ANS* genes inhibit the synthesis of anthocyanins, thus resulting in the formation of white flowers. According to the data of metabolomics and transcriptomics, the low expression of *SbDFR* reduced the anthocyanin levels in *S*. *baicalensis* flowers, and the differential expression of *Sb3GT* led to the formation of purple flower and purple-red flower of *S*. *baicalensis*.

### Function of *SbDFR* and *Sb3GT* Genes

Anthocyanin synthesis in plants is affected by a series of key enzyme genes and related transcription factors (Suzuki et al., 2016). It has been reported that the down-regulation of *DFR* and *ANS* genes in the anthocyanin synthesis pathway leads to pigmentation loss (Bogs et al., 2007; Clark and Verwoerd, 2011). The over-expression of different *DFR* genes in tobacco flowers promotes the biosynthesis of anthocyanins and increases the deposition of red pigments (Luo et al., 2016). Our study demonstrated that the white-flowered characteristic was attributed to the down-regulated expression of *DFR*. Anthocyanins are highly unstable and easily degraded, so glycosylation is very important to stabilize anthocyanins. 3GT belongs to the flavonoid glycosyltransferase family and has a highly conserved PSPG box. It can catalyze the modification of unstable anthocyanins in plants and transform them into stable anthocyanins (Wu et al., 2017). The 3GT gene of plum blossom has higher activity in red flowers than in white flowers, and the enzyme activity increases with the appearance of red coloring (Niu et al., 2010). The accumulation of anthocyanins in *Myrica rubra* fruit is related to the coordinated expression of many biosynthetic genes (DFR, ANS, F3′H, F3H, and 3GT) and is regulated by MrMYB1 (Wu et al., 2017). Our study identified two *Sb3GT* genes (*Sb07g04780 and Sb01g72290*), and the differential expression of *Sb3GT* led to the formation of purple and purple-red flowers in *S*. *baicalensis*. However, we speculated that the *Sb3GT* (Sb01g72290) gene is a new 3GT gene in *S*. *baicalensis*, which functions in purple and purple-red flowers in *S*. *baicalensis* and needs further study.

### Transcription Factors

Studies have shown that TFs play an essential role in the modulation of anthocyanin synthesis pathways. For example, FtMYB1 and FtMYB2 in tartary buckwheat and VvMYBA2 in grapes can promote the expression of *DFR* (Bai et al., 2014; Niu et al., 2016). In apples, the *CHS* gene is positively correlated with the expression of MYB4 and MYB5 (Clark and Verwoerd, 2011). Abnormal expression of bHLH3 disrupts the balance of the flavonoid regulatory network required for fruit development, leading to differences in pigment composition in mulberry fruit (Lloyd et al., 2017). MYB TFs (e.g., PAP1/PAP2, TT2, MYB-75, -90, -113, and 114), bHLH TFs (e.g., EGL3, GL3, and TT8), and WD40 repeat protein (TTG1) in *Arabidopsis thaliana* form an MBW complex to participate in regulating the expression of *UFGT*, *ANS*, *DFR*, and other downstream genes, thus affecting the biosynthesis of anthocyanins (Xu et al., 2014). Apart from MYB, bHLH, and WD40, transcription factors of zinc fingers, MADs, and WRKY proteins are also responsible for regulating the biosynthesis of anthocyanins (Terrier et al., 2009; Li H. et al., 2020). Besides, *IbMADS10* modulates the biosynthesis of anthocyanins to enhance anthocyanin pigment accumulation in sweet potato (Lalusin et al., 2011). NAC TFs (e.g., CUC2, ATAF-1, -2, and NAM) have shown to regulates biosynthesis of anthocyanins in blood-fleshed peaches (Zhou et al., 2015). It has been reported that MYB114 and MYB75 play a key role in the modulation of anthocyanin synthesis pathways. Suppression of PyMYB114 could inhibit anthocyanin biosynthesis in red-skinned pears (Yao et al., 2017). Co-transformation of Pp12ERF96 with PpMYB114 and PpbHLH3 in tobacco leaves led to enhanced anthocyanin accumulation (Ni et al., 2019). MYB75 defined as PRODUCTION OF ANTHOCYANIN PIGMENT 1 (PAP1), plays a key role in anthocyanin accumulation (Antonio et al., 2008). In this study, after analysis and comparison of the *Scutellaria baicalensis* petal transcriptome sequencing database, no related genes with high homology of MYB114 and MYB75 were found, but we found that myb family transcription factor APL isoform X3 (Supplementary Table 6) was significantly positively correlated with the accumulation of anthocyanins. It is speculated that myb family transcription factor APL isoform X3 can play a key role in the synthesis of anthocyanin of *Scutellaria baicalensis*, which its regulatory role may similar to the reported TFs of MYB114 and myb75, but the specific function needs to be further verified by more experiments.

In our study, 127, 79, and 73 TFs-related DEGs were identified in SbP vs. SbW, SbP vs. SbPR, and SbPR vs. SbW, respectively, including bHLH, MYB, WRKY, WD40, NACs, and MADS-box (Supplementary Table 8). We speculate that these TFs-related DEGs (such as WRKY, bHLH, and NAC) can serve as key players of anthocyanin synthesis in *S*. *baicalensis* flowers. Correlation analysis also revealed that the TFs WRKY, bHLH, and NAC were closely related to anthocyanin accumulation, gene expression and anthocyanin synthesis in different degree of *S*. *baicalensis* flowers, but their patterns of regulating gene expression and metabolite biosynthesis are unclear. Therefore, further studies are needed to verify whether these candidate TFs can form a ternary protein complex or alone directly affects anthocyanin synthesis in *S*. *baicalensis* flowers.

## Conclusion

To elucidate the underlying mechanism of color differentiation in metabolic and transcriptional differences among three kinds of *S*. *baicalensis* flowers, metabolic, transcriptional, and integration analyses were performed, and the DEGs responsible for the biosynthesis and regulation of anthocyanins were identified. Our results showed that cyanidin 3-rutin and delphinium chloride metabolites were considered to be the key anthocyanins for pigment accumulation in *S*. *baicalensis* flowers, and the expression levels of *SbDFR* and *Sb3GT* genes were strongly correlated with the anthocyanin biosynthesis process in *S*. *baicalensis* flowers. In addition, transcription factors such as WRKY, bHLH, and NAC, especially for NAC35, which were also positively involved in anthocyanin biosynthesis in *S*. *baicalensis* flowers. Our results will provide a solid foundation for understanding the roles of DEGs and TFs in regulating anthocyanin biosynthesis in *S*. *baicalensis* flowers and also guide future directional breeding of new *S*. *baicalensis* germplasm for enriched agricultural landscape design.

## Data Availability Statement

The datasets presented in this study can be found in online repositories. The names of the repository/repositories and accession number(s) can be found below: National Center for Biotechnology Information (NCBI) BioProject database under accession number PRJNA811392; https://www.ncbi.nlm.nih.gov/bioproject/PRJNA811392.

## Author Contributions

DW, JW, and YN conceived and designed the experiments. JW and YW collected the plant samples. JW, DY, and YW performed the experiments. DY conducted bioinformatics analysis. DW and JW wrote the manuscript. All authors read and approved the final manuscript.

## Conflict of Interest

The authors declare that the research was conducted in the absence of any commercial or financial relationships that could be construed as a potential conflict of interest.

## Publisher’s Note

All claims expressed in this article are solely those of the authors and do not necessarily represent those of their affiliated organizations, or those of the publisher, the editors and the reviewers. Any product that may be evaluated in this article, or claim that may be made by its manufacturer, is not guaranteed or endorsed by the publisher.

## Footnotes

1
http://www.anpel.com.cn/

2
www.shimadzu.com.cn/

3
www.appliedbiosystems.com.cn/

4
http://www.massbank.jp/

5
http://www.knapsackfamily.com/KNApSAcK/

6
http://www.hmdb.ca/

7
https://ngdc.cncb.ac.cn/databasecommons/database/id/3091

8
http://metlin.scripps.edu/index.php

9
www.r-project.org/

10
http://www.genome.jp/kegg

11
https://www.omicstudio.cn/tool/64

12
http://zhanglab.ccmb.med.umich.edu/I-TASSER/

13
https://www.cgl.ucsf.edu/chimera/

## Supplementary Material

Supplementary Figure 1 The heatmap analysis of all differentially accumulated metabolites among the flowers of SbW, SbPR, and SbP. The color scale on the right represents the expression degree of differentially accumulated flavonoid metabolites in the material. Red represents high accumulation level and blue represents low accumulation level.

Supplementary Figure 2 The heatmap analysis of all differentially accumulated anthocyanins among the flowers of SbW, SbPR, and SbP according to the relative content in samples. The color scale on the right represents the expression degree of differentially accumulated anthocyanin metabolites in the material. Red represents high accumulation level and blue represents low accumulation level.

Supplementary Figure 3 Number of differentially accumulated anthocyanins among SbPR vs SbP, SbW vs SbP, and SbW vs SbPR.
