# Supplementary figures and images for: Metabolomic and Transcriptomic Profiling Uncover the Underlying Mechanism of Color Differentiation in Scutellaria baicalensis Georgi. Flowers
Source: Front Plant Sci. 2022 Jun 9;13:884957. doi: 10.3389/fpls.2022.884957 (PMC9218823; doi:10.3389/fpls.2022.884957)

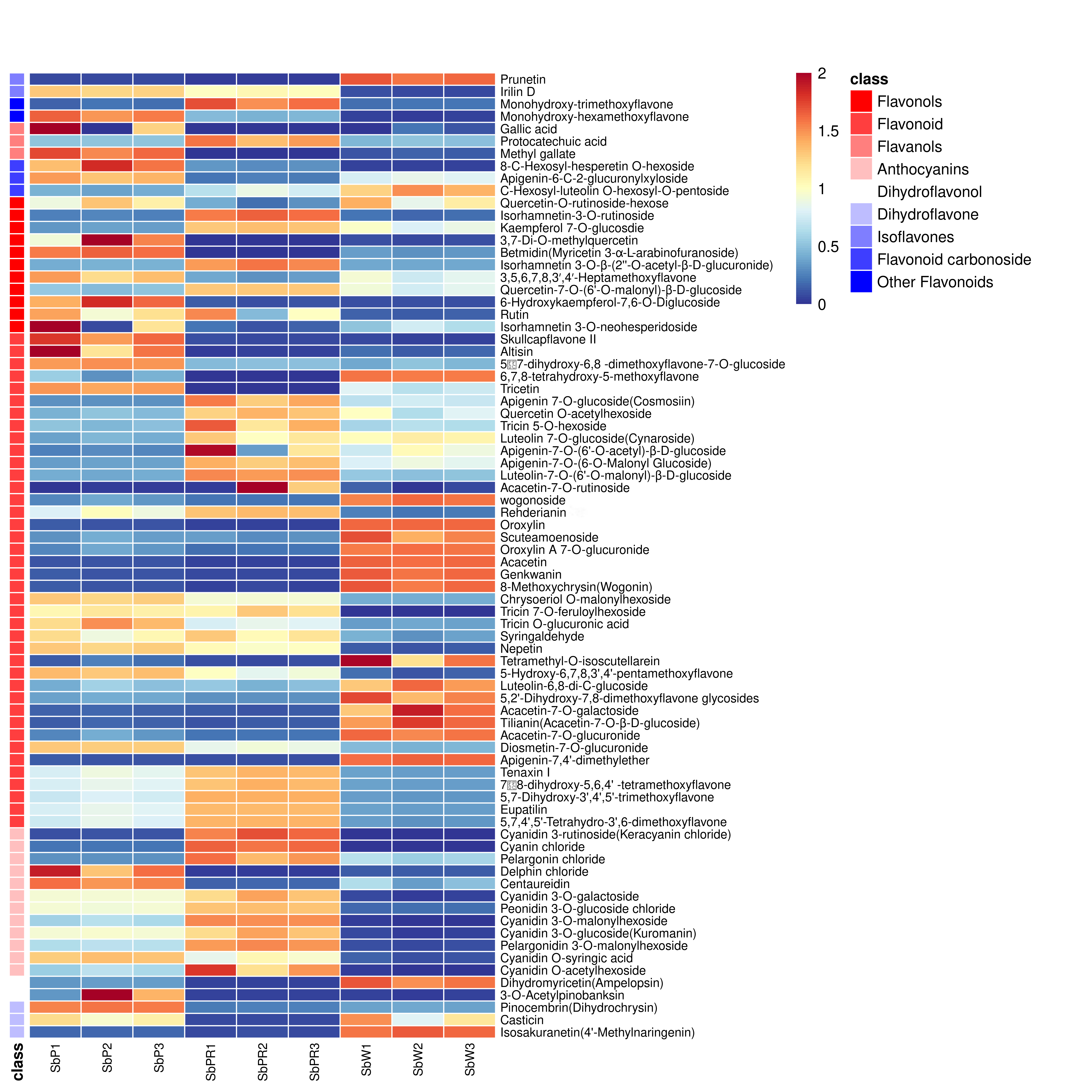

Supplement: Supplementary Figure 1 — The heatmap analysis of all differentially accumulated metabolites among the flowers of SbW, SbPR, and SbP. The color scale on the right represents the expression degree of differentially accumulated flavonoid metabolites in the material. Red represents high accumulation level and blue represents low accumulation level. [file Image_1.jpeg]

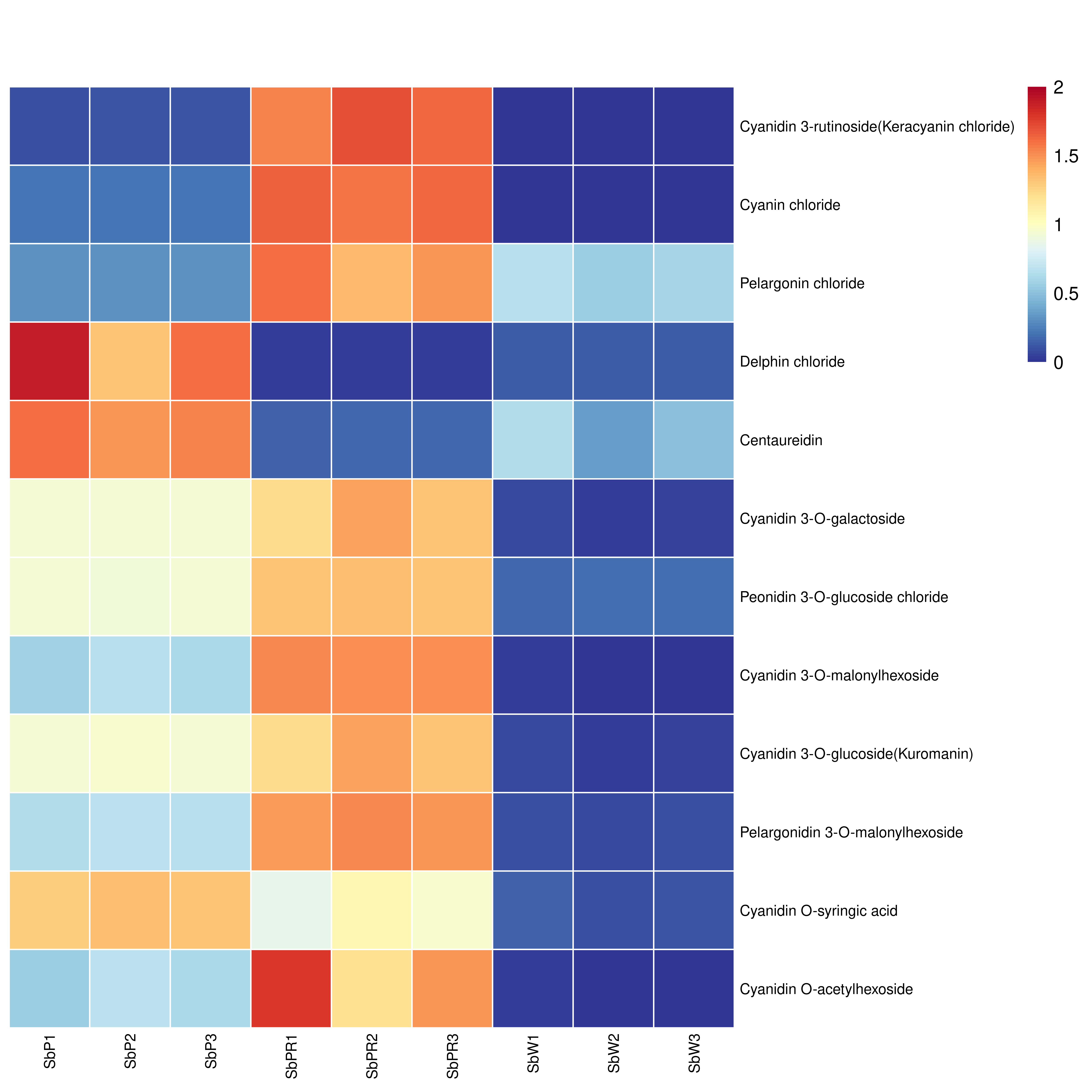

Supplement: Supplementary Figure 2 — The heatmap analysis of all differentially accumulated anthocyanins among the flowers of SbW, SbPR, and SbP according to the relative content in samples. The color scale on the right represents the expression degree of differentially accumulated anthocyanin metabolites in the material. Red represents high accumulation level and blue represents low accumulation level. [file Image_2.jpeg]

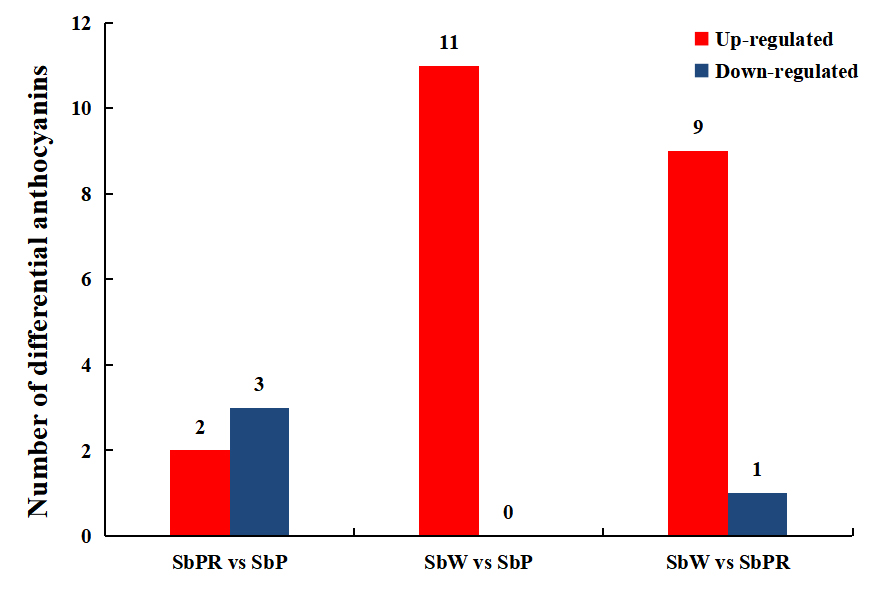

Supplement: Supplementary Figure 3 — Number of differentially accumulated anthocyanins among SbPR vs SbP, SbW vs SbP, and SbW vs SbPR. [file Image_3.jpeg]
